# Supplementary figures and images for: Eight single nucleotide polymorphisms and their association with food habit domestication traits and growth traits in largemouth bass fry (Micropterus salmoides) based on PCR-RFLP method
Source: PeerJ. 2023 Jan 9;11:e14588. doi: 10.7717/peerj.14588 (PMC9835702; doi:10.7717/peerj.14588)

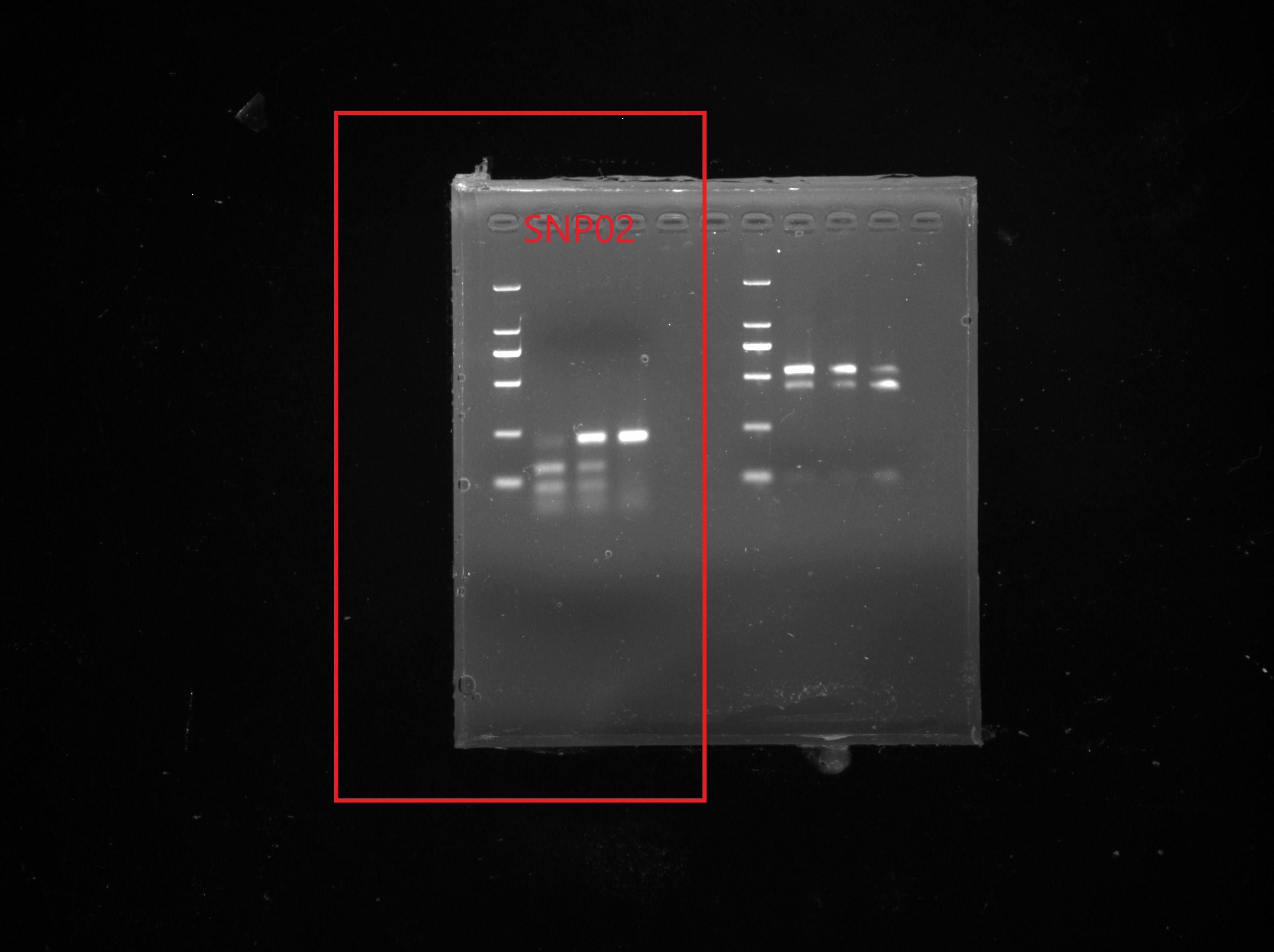

Supplement: Supplemental Information 1 — Red box marked specific SNPs. [file peerj-11-14588-s001.zip › row figure/SNP02.jpg]

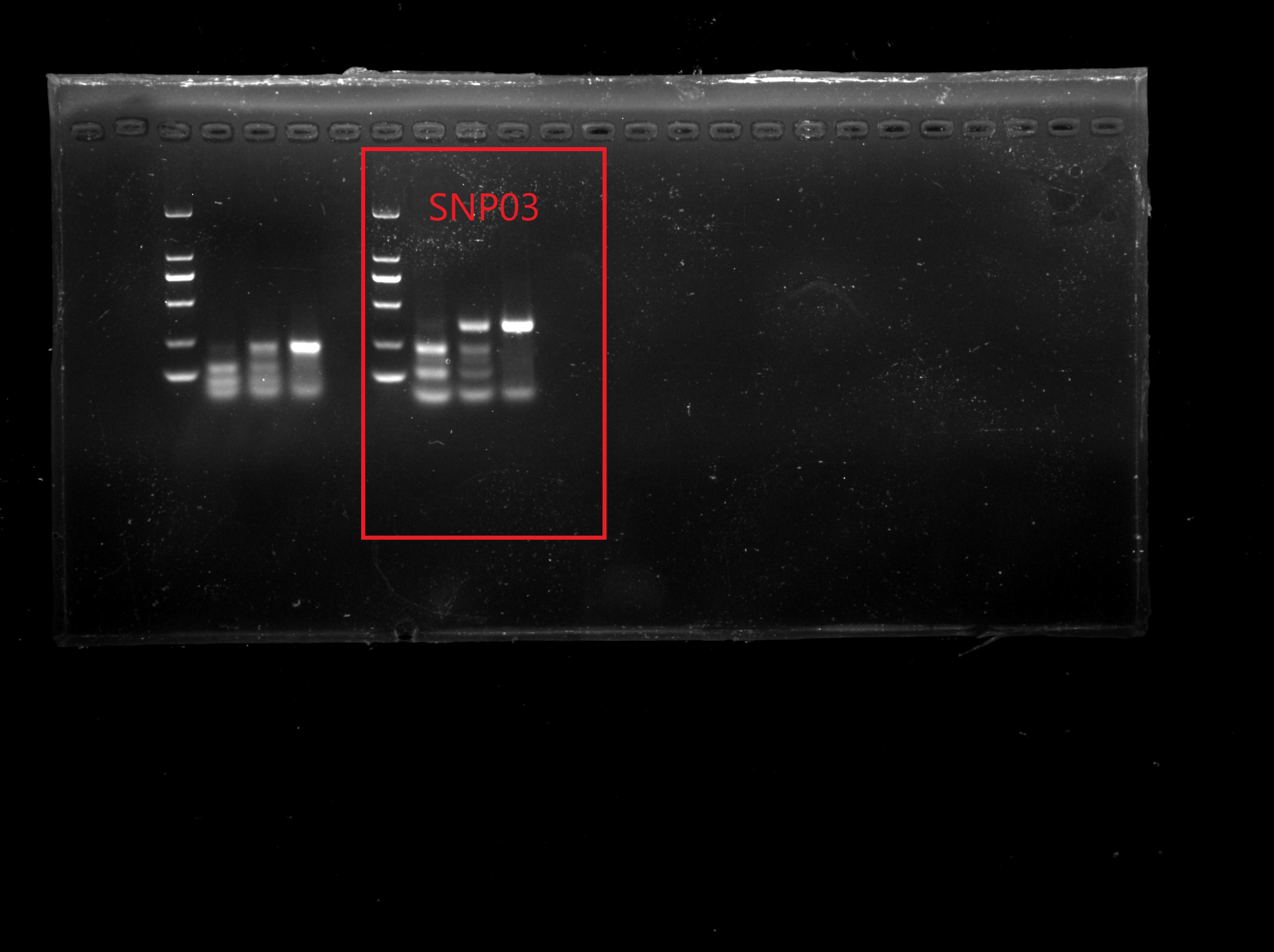

Supplement: Supplemental Information 1 — Red box marked specific SNPs. [file peerj-11-14588-s001.zip › row figure/SNP03.jpg]

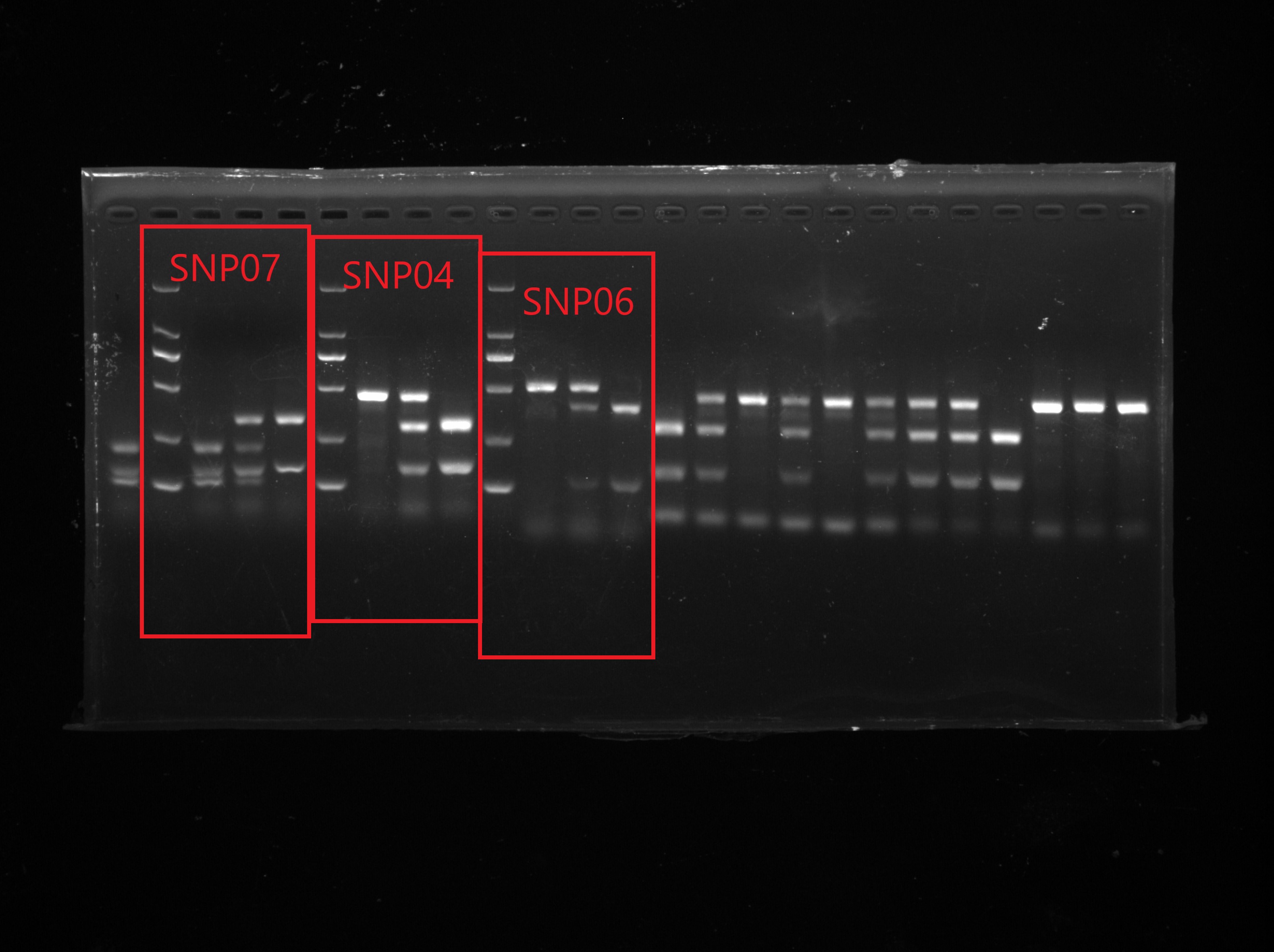

Supplement: Supplemental Information 1 — Red box marked specific SNPs. [file peerj-11-14588-s001.zip › row figure/SNP07íóSNP04&SNP06.jpg]

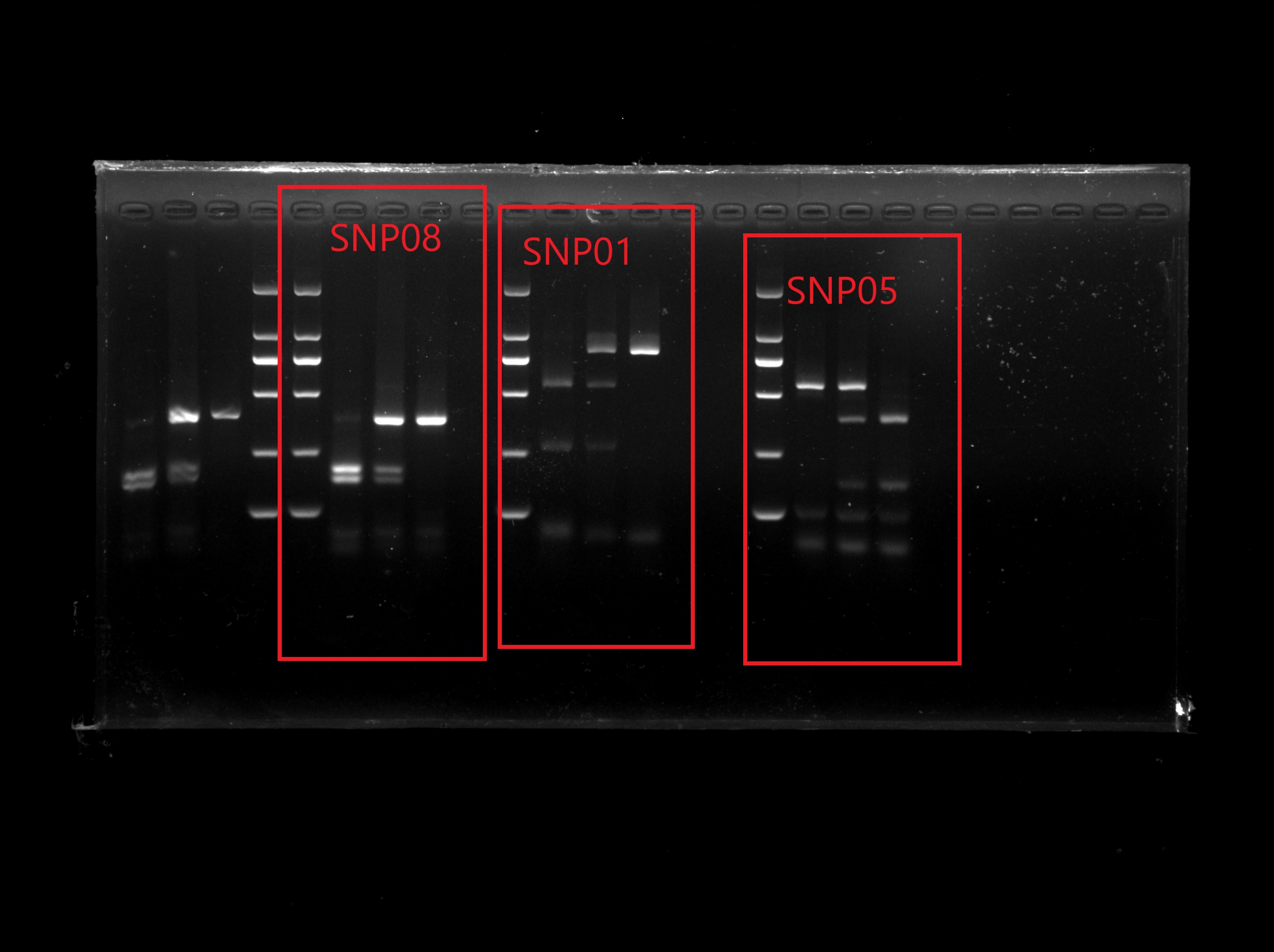

Supplement: Supplemental Information 1 — Red box marked specific SNPs. [file peerj-11-14588-s001.zip › row figure/SNP08íóSNP01&SNP05.jpg]
