# Supplementary material for: Eight single nucleotide polymorphisms and their association with food habit domestication traits and growth traits in largemouth bass fry (Micropterus salmoides) based on PCR-RFLP method
Source: PeerJ. 2023 Jan 9;11:e14588. doi: 10.7717/peerj.14588 (PMC9835702; doi:10.7717/peerj.14588)
Supplement: Supplemental Information 3 — Basic calculation process [file peerj-11-14588-s003.docx]

**Table 1.** Primer information of eight SNPs in juvenile *M. salmoides*

No statistical analysis.

**Table 2** Genotype frequency and genetic parameters of eight SNPs in juvenile *M. salmoides*

Microsoft Excel software was used for statistical analysis of morphological data and genotyping results. Genotype frequency equal to number divided by total (192). The analyses of the observed heterozygosity (Ho), expected heterozygosity (He), and the polymorphic information content (PIC) were performed using Cervus 3.0 software. Popgen32 software was used to analyze the Hardy-Weinberg equilibrium.

**SNP01**

Chi-square test for Hardy-Weinberg equilibrium :

Chi-square : 0.001565

Degree of freedom : 1

Probability : 0.968444

**SNP02**

Chi-square test for Hardy-Weinberg equilibrium :

Chi-square : 2.488908

Degree of freedom : 1

Probability : 0.114651

**SNP03**

Chi-square test for Hardy-Weinberg equilibrium :

Chi-square : 4.890469

Degree of freedom : 1

Probability : 0.027005

**SNP04**

Chi-square test for Hardy-Weinberg equilibrium :

Chi-square : 1.093600

Degree of freedom : 1

Probability : 0.295675

**SNP05**

Chi-square test for Hardy-Weinberg equilibrium :

Chi-square : 1.220975

Degree of freedom : 1

Probability : 0.269169

**SNP06**

Chi-square test for Hardy-Weinberg equilibrium :

Chi-square : 0.024735

Degree of freedom : 1

Probability : 0.875029

**SNP07**

Chi-square test for Hardy-Weinberg equilibrium :

Chi-square : 0.949828

Degree of freedom : 1

Probability : 0.329763

**SNP08**

Chi-square test for Hardy-Weinberg equilibrium :

Chi-square : 1.305086

Degree of freedom : 1

Probability : 0.253286

**Table 3.** Correlation analysis between eight SNPs and food habit domestication traits in juvenile *M. salmoides*

Microsoft Excel software was used for statistical analysis of morphological data and genotyping results. The correlation between genotypes at each locus and food habit domestication traits of juvenile fish was analyzed using the chi-square or Fisher exact test in R software. Specific data are as follows:

1-96: Domesticated 97-192: Non-domesticated

**SNP01**

GG 59 35

GA 33 48

AA 4 13

yx<-matrix(c(59,33,4,35,48,13), nrow=3, ncol=2)

chisq.test(yx)

X-squared=13.67, df=2, p-value=0.001075

**SNP02**

TT 11 7

TA 41 26

AA 44 63

yx<-matrix(c(11,41,44,7,26,63), nrow=3, ncol=2)

chisq.test(yx)

X-squared=7.6209, df=2, p-value=0.02214

**SNP03**

GG 14 5

GA 49 55

AA 33 36

yx<-matrix(c(14,49,33,5,55,36), nrow=3, ncol=2)

chisq.test(yx)

X-squared=4.7397, df=2, p-value=0.09349

**SNP04**

TT 28 48

TC 53 42

CC 15 6

yx<-matrix(c(28,53,15,48,42,6), nrow=3, ncol=2)

chisq.test(yx)

X-squared=10.394, df=2, p-value=0.005533

**SNP05**

GG 34 24

GC 42 60

CC 20 12

yx<-matrix(c(34,42,20,24,60,12), nrow=3, ncol=2)

chisq.test(yx)

X-squared=6.9006, df=2, p-value=0.03174

**SNP06**

CC 47 28

CT 38 53

TT 11 15

yx<-matrix(c(47,38,11,28,53,15), nrow=3, ncol=2)

chisq.test(yx)

X-squared=7.9012, df=2, p-value=0.01924

**SNP07**

CC 19 26

CA 49 54

AA 28 16

yx<-matrix(c(19,49,28,26,54,16), nrow=3, ncol=2)

chisq.test(yx)

X-squared=4.6043, df=2, p-value=0.1

**SNP08**

CC 54 58

CA 36 37

AA 6 1

yx<-matrix(c(54,36,6,58,37,1), nrow=3, ncol=2)

chisq.test(yx)

X-squared=3.728, df=2, p-value=0.1551

The Chi-squared approximation algorithm may not be accurate

Fisher.test(yx)

p-value=0.2018

**Table 4** Effect of body length and body height traits on body weight in juvenile *M. salmoides*

SPSS22 software (IBM Software, USA) was used for the logarithmic transformation of growth traits, phenotypic correlation analysis, and weight path analysis.

1. normal distribution test

|  | Kolmogorov-Smirnov^a^ | | | Shapiro-Wilk | | |
| --- | --- | --- | --- | --- | --- | --- |
|  | Statistic | df | Sig. | Statistic | df | Sig. |
| Body length | 0.051 | 192 | 0.200 | 0.989 | 192 | 0.134 |
| Body height | 0.92 | 192 | 0.000 | 0.960 | 192 | 0.000 |
| Body weight | 0.114 | 192 | 0.000 | 0.976 | 192 | 0.000 |

Sig.>0.05, conform to normal distribution

1. logarithmic transformation
2. linear regression

| Traits | Correlation Coefficient | Sig. |
| --- | --- | --- |
| Body Length | 0.840 | 0.000 |
| Body Height | 0.842 | 0.000 |

**Table 5** Correlation analysis between the different genotypes of eight SNPs and growth traits in juvenile *M. salmoides*

Microsoft Excel software was used for statistical analysis of morphological data and genotyping results. The general linear model in SPSS22 software was used to analyze the correlation between the genotypes at each locus and body weight, height, and length of juvenile largemouth bass. Specific data are as follows:

**SNP01** genotype number

GG 94

GA 81

AA 17

Statistical data: mean ± standard error of the mean

Body Weight(mg) Body Height(mm) Body Length(mm)

0.100±0.003 5.644±0.074 20.638±0.200

0.091±0.003 5.390±0.064 20.109±0.178

0.079±0.005 4.969±0.148 19.770±0.358

Analyze-Compare means-One-way ANOVA

analysis method-LSD significance level=0.05

| Length | Genotype | Genotype | Significance | 95% Confidence Interval | |
| --- | --- | --- | --- | --- | --- |
|  |  |  |  | Lower Bound | Upper Bound |
| LSD | AA | GA | 0.47 | -1.27 | 0.59 |
|  |  | GG | 0.06 | -1.79 | 0.05 |
|  | GA | AA | 0.47 | -0.59 | 1.27 |
|  |  | GG | 0.05 | -1.06 | -0.00 |
|  | GG | AA | 0.06 | 0.05 | 1.79 |
|  |  | GA | 0.05 | 0.00 | 1.06 |

| Weight | Genotype | Genotype | Significance | 95% Confidence Interval | |
| --- | --- | --- | --- | --- | --- |
|  |  |  |  | Lower Bound | Upper Bound |
| LSD | AA | GA | 0.02 | -0.76 | -0.08 |
|  |  | GG | 0.00 | -1.01 | -0.34 |
|  | GA | AA | 0.02 | 0.08 | 0.76 |
|  |  | GG | 0.01 | -0.45 | -0.06 |
|  | GG | AA | 0.00 | 0.34 | 1.01 |
|  |  | GA | 0.01 | 0.06 | 0.45 |

| Height | Genotype | Genotype | Significance | 95% Confidence Interval | |
| --- | --- | --- | --- | --- | --- |
|  |  |  |  | Lower Bound | Upper Bound |
| LSD | AA | GA | 0.09 | -0.02 | -0.00 |
|  |  | GG | 0.00 | -0.03 | -0.01 |
|  | GA | AA | 0.09 | -0.00 | 0.02 |
|  |  | GG | 0.01 | -0.02 | -0.00 |
|  | GG | AA | 0.00 | 0.01 | 0.03 |
|  |  | GA | 0.01 | 0.00 | 0.02 |

**SNP02** genotype number

TT 18

TA 67

AA 107

Statistical data: mean ± standard error of the mean

Body Weight(mg) Body Height(mm) Body Length(mm)

0.091±0.006 5.422±0.166 20.283±0.431

0.099±0.003 5.532±0.083 20.618±0.209

0.093±0.002 5.452±0.065 20.172±0.176

Analyze-Compare means-One-way ANOVA

analysis method-LSD significance level=0.05

| Length | Genotype | Genotype | Significance | 95% Confidence Interval | |
| --- | --- | --- | --- | --- | --- |
|  |  |  |  | Lower Bound | Upper Bound |
| LSD | TT | TA | 0.48 | -1.27 | 0.60 |
|  |  | AA | 0.81 | -0.78 | 1.01 |
|  | TA | TT | 0.48 | -0.60 | 1.27 |
|  |  | AA | 0.11 | -0.10 | 0.99 |
|  | AA | TT | 0.81 | -1.01 | 0.78 |
|  |  | TA | 0.11 | -0.99 | 0.10 |

| Weight | Genotype | Genotype | Significance | 95% Confidence Interval | |
| --- | --- | --- | --- | --- | --- |
|  |  |  |  | Lower Bound | Upper Bound |
| LSD | TT | TA | 0.54 | -0.47 | 0.25 |
|  |  | AA | 0.86 | -0.37 | 0.31 |
|  | TA | TT | 0.54 | -0.25 | 0.47 |
|  |  | AA | 0.45 | -0.13 | 0.29 |
|  | AA | TT | 0.86 | -0.31 | 0.37 |
|  |  | TA | 0.45 | -0.29 | 0.13 |

| Height | Genotype | Genotype | Significance | 95% Confidence Interval | |
| --- | --- | --- | --- | --- | --- |
|  |  |  |  | Lower Bound | Upper Bound |
| LSD | TT | TA | 0.24 | -0.02 | 0.01 |
|  |  | AA | 0.80 | -0.01 | 0.01 |
|  | TA | TT | 0.24 | -0.01 | 0.02 |
|  |  | AA | 0.11 | -0.00 | 0.01 |
|  | AA | TT | 0.80 | -0.01 | 0.01 |
|  |  | TA | 0.11 | -0.01 | 0.01 |

**SNP03** genotype number

GG 19

GA 104

AA 69

Statistical data: mean ± standard error of the mean

Body Weight(mg) Body Height(mm) Body Length(mm)

0.104±0.005 5.734±0.143 20.692±0.288

0.093±0.002 5.439±0.064 20.347±0.175

0.094±0.003 5.464±0.088 20.227±0.230

Analyze-Compare means-One-way ANOVA

analysis method-LSD significance level=0.05

| Length | Genotype | Genotype | Significance | 95% Confidence Interval | |
| --- | --- | --- | --- | --- | --- |
|  |  |  |  | Lower Bound | Upper Bound |
| LSD | GG | GA | 0.44 | -0.53 | 1.22 |
|  |  | AA | 0.31 | -0.45 | 1.38 |
|  | GA | GG | 0.44 | -1.22 | 0.54 |
|  |  | AA | 0.66 | -0.43 | 0.67 |
|  | AA | GG | 0.32 | -1.38 | 0.45 |
|  |  | GA | 0.66 | -0.67 | 0.43 |

| Weight | Genotype | Genotype | Significance | 95% Confidence Interval | |
| --- | --- | --- | --- | --- | --- |
|  |  |  |  | Lower Bound | Upper Bound |
| LSD | GG | GA | 0.08 | -0.04 | 0.63 |
|  |  | AA | 0.13 | -0.08 | 0.62 |
|  | GA | GG | 0.08 | -0.63 | 0.04 |
|  |  | AA | 0.81 | -0.23 | 0.18 |
|  | AA | GG | 0.13 | -0.62 | 0.08 |
|  |  | GA | 0.81 | -0.18 | 0.23 |

| Height | Genotype | Genotype | Significance | 95% Confidence Interval | |
| --- | --- | --- | --- | --- | --- |
|  |  |  |  | Lower Bound | Upper Bound |
| LSD | GG | GA | 0.08 | -0.00 | 0.02 |
|  |  | AA | 0.12 | -0.00 | 0.02 |
|  | GA | GG | 0.08 | -0.02 | 0.00 |
|  |  | AA | 0.81 | -0.01 | 0.01 |
|  | AA | AA | 0.12 | -0.02 | 0.00 |
|  |  | GA | 0.81 | -0.01 | 0.01 |

**SNP04** genotype number

TT 76

TC 95

CC 21

Statistical data: mean ± standard error of the mean

Body Weight(mg) Body Height(mm) Body Length(mm)

0.094±0.003 5.394±0.080 20.448±0.199

0.096±0.003 5.546±0.070 20.353±0.190

0.091±0.005 5.467±0.128 19.868±0.365

Analyze-Compare means-One-way ANOVA

analysis method-LSD significance level=0.05

| Length | Genotype | Genotype | Significance | 95% Confidence Interval | |
| --- | --- | --- | --- | --- | --- |
|  |  |  |  | Lower Bound | Upper Bound |
| LSD | TT | TC | 0.73 | -0.45 | 0.64 |
|  |  | CC | 0.19 | -0.29 | 1.45 |
|  | TC | TT | 0.73 | -0.63 | 0.45 |
|  |  | CC | 0.26 | -0.36 | 1.33 |
|  | CC | TT | 0.19 | -1.45 | 0.29 |
|  |  | TC | 0.26 | -1.33 | 0.36 |

| Weight | Genotype | Genotype | Significance | 95% Confidence Interval | |
| --- | --- | --- | --- | --- | --- |
|  |  |  |  | Lower Bound | Upper Bound |
| LSD | TT | TC | 0.15 | -0.36 | 0.05 |
|  |  | CC | 0.67 | -0.40 | 0.26 |
|  | TC | TT | 0.15 | -0.05 | 0.36 |
|  |  | CC | 0.63 | -0.24 | 0.40 |
|  | CC | TT | 0.67 | -0.26 | 0.40 |
|  |  | TC | 0.63 | -0.40 | 0.24 |

| Height | Genotype | Genotype | Significance | 95% Confidence Interval | |
| --- | --- | --- | --- | --- | --- |
|  |  |  |  | Lower Bound | Upper Bound |
| LSD | TT | TC | 0.53 | -0.01 | 0.01 |
|  |  | CC | 0.66 | -0.01 | 0.02 |
|  | TC | TT | 0.53 | -0.01 | 0.01 |
|  |  | CC | 0.39 | -0.01 | 0.02 |
|  | CC | TT | 0.66 | -0.02 | 0.01 |
|  |  | TC | 0.39 | -0.02 | 0.01 |

**SNP05** genotype number

GG 58

GC 102

CC 32

Statistical data: mean ± standard error of the mean

Body Weight(mg) Body Height(mm) Body Length(mm)

0.097±0.003 5.505±0.089 20.458±0.237

0.095±0.003 5.467±0.071 20.458±0.179

0.089±0.004 5.460±0.099 19.772±0.285

Analyze-Compare means-One-way ANOVA

analysis method-LSD significance level=0.05

| Length | Genotype | Genotype | Significance | 95% Confidence Interval | |
| --- | --- | --- | --- | --- | --- |
|  |  |  |  | Lower Bound | Upper Bound |
| LSD | GG | GC | 0.97 | -0.56 | 0.59 |
|  |  | CC | 0.09 | -0.08 | 1.46 |
|  | GC | GG | 0.97 | -0.59 | 0.56 |
|  |  | CC | 0.06 | -0.03 | 1.38 |
|  | CC | GG | 0.09 | -1.46 | 0.08 |
|  |  | GC | 0.06 | -1.38 | 0.03 |

| Weight | Genotype | Genotype | Significance | 95% Confidence Interval | |
| --- | --- | --- | --- | --- | --- |
|  |  |  |  | Lower Bound | Upper Bound |
| LSD | GG | GC | 0.74 | -0.18 | 0.26 |
|  |  | CC | 0.77 | -0.25 | 0.34 |
|  | GC | GG | 0.74 | -0.26 | 0.18 |
|  |  | CC | 0.96 | -0.26 | 0.28 |
|  | CC | GG | 0.77 | -0.34 | 0.25 |
|  |  | GC | 0.96 | -0.28 | 0.26 |

| Height | Genotype | Genotype | Significance | 95% Confidence Interval | |
| --- | --- | --- | --- | --- | --- |
|  |  |  |  | Lower Bound | Upper Bound |
| LSD | GG | GC | 0.67 | -0.01 | 0.01 |
|  |  | CC | 0.14 | -0.00 | 0.02 |
|  | GC | GG | 0.67 | -0.01 | 0.01 |
|  |  | CC | 0.20 | -0.00 | 0.02 |
|  | CC | GG | 0.14 | -0.02 | 0.00 |
|  |  | GC | 0.20 | -0.02 | 0.00 |

**SNP06** genotype number

CC 75

CT 91

TT 26

Statistical data: mean ± standard error of the mean

Body Weight(mg) Body Height(mm) Body Length(mm)

0.093±0.003 5.469±0.083 20.082±0.213

0.097±0.003 5.533±0.068 20.571±0.182

0.090±0.005 5.308±0.132 20.260±0.340

Analyze-Compare means-One-way ANOVA

analysis method-LSD significance level=0.05

| Length | Genotype | Genotype | Significance | 95% Confidence Interval | |
| --- | --- | --- | --- | --- | --- |
|  |  |  |  | Lower Bound | Upper Bound |
| LSD | CC | CT | 0.08 | -1.04 | 0.06 |
|  |  | TT | 0.66 | -0.98 | 0.62 |
|  | CT | CC | 0.08 | -0.06 | 1.04 |
|  |  | TT | 0.43 | -0.47 | 1.10 |
|  | TT | TT | 0.66 | -0.62 | 0.98 |
|  |  | TC | 0.43 | -1.10 | 0.47 |

| Weight | Genotype | Genotype | Significance | 95% Confidence Interval | |
| --- | --- | --- | --- | --- | --- |
|  |  |  |  | Lower Bound | Upper Bound |
| LSD | CC | CT | 0.55 | -0.27 | 0.14 |
|  |  | TT | 0.30 | -0.14 | 0.47 |
|  | CT | CC | 0.55 | -0.14 | 0.27 |
|  |  | TT | 0.14 | -0.07 | 0.52 |
|  | TT | TT | 0.30 | -0.47 | 0.14 |
|  |  | CT | 0.14 | -0.52 | 0.07 |

| Height | Genotype | Genotype | Significance | 95% Confidence Interval | |
| --- | --- | --- | --- | --- | --- |
|  |  |  |  | Lower Bound | Upper Bound |
| LSD | CC | CT | 0.33 | -0.01 | 0.00 |
|  |  | TT | 0.60 | -0.01 | 0.01 |
|  | CT | CC | 0.33 | -0.00 | 0.01 |
|  |  | TT | 0.22 | -0.00 | 0.02 |
|  | TT | CC | 0.60 | -0.01 | 0.01 |
|  |  | CT | 0.22 | -0.02 | 0.00 |

**SNP07** genotype number

CC 45

CA 103

AA 44

Statistical data: mean ± standard error of the mean

Body Weight(mg) Body Height(mm) Body Length(mm)

0.091±0.003 5.312±0.093 20.138±0.226

0.095±0.003 5.489±0.069 20.355±0.181

0.098±0.004 5.618±0.100 20.503±0.292

Analyze-Compare means-One-way ANOVA

analysis method-LSD significance level=0.05

| Length | Genotype | Genotype | Significance | 95% Confidence Interval | |
| --- | --- | --- | --- | --- | --- |
|  |  |  |  | Lower Bound | Upper Bound |
| LSD | CC | CA | 0.50 | -0.85 | 0.41 |
|  |  | AA | 0.34 | -1.11 | 0.38 |
|  | CA | CC | 0.50 | -0.41 | 0.85 |
|  |  | AA | 0.65 | -0.78 | 0.49 |
|  | AA | CC | 0.34 | -0.38 | 1.11 |
|  |  | CA | 0.65 | -0.49 | 0.78 |

| Weight | Genotype | Genotype | Significance | 95% Confidence Interval | |
| --- | --- | --- | --- | --- | --- |
|  |  |  |  | Lower Bound | Upper Bound |
| LSD | CC | CA | 0.14 | -0.41 | 0.06 |
|  |  | AA | 0.03 | -0.59 | 0.02 |
|  | CA | CC | 0.14 | -0.06 | 0.41 |
|  |  | AA | 0.29 | -0.37 | 0.11 |
|  | AA | CC | 0.03 | -0.02 | 0.59 |
|  |  | CA | 0.29 | -0.11 | 0.37 |

| Height | Genotype | Genotype | Significance | 95% Confidence Interval | |
| --- | --- | --- | --- | --- | --- |
|  |  |  |  | Lower Bound | Upper Bound |
| LSD | CC | CA | 0.36 | -0.01 | 0.00 |
|  |  | AA | 0.20 | -0.02 | 0.00 |
|  | CA | CC | 0.36 | -0.00 | 0.01 |
|  |  | AA | 0.54 | -0.01 | 0.01 |
|  | AA | CC | 0.20 | -0.0 | 0.02 |
|  |  | CA | 0.54 | -0.01 | 0.01 |

**SNP08** genotype number

CC 112

CA 73

AA 7

Statistical data: mean ± standard error of the mean

Body Weight(mg) Body Height(mm) Body Length(mm)

0.094±0.002 5.473±0.065 20.273±0.156

0.095±0.003 5.463±0.080 20.418±0.234

0.103±0.010 5.699±0.191 20.547±0.664

Analyze-Compare means-One-way ANOVA

analysis method-LSD significance level=0.05

| Length | Genotype | Genotype | Significance | 95% Confidence Interval | |
| --- | --- | --- | --- | --- | --- |
|  |  |  |  | Lower Bound | Upper Bound |
| LSD | CC | CA | 0.59 | -0.68 | 0.39 |
|  |  | AA | 0.70 | -1.65 | 1.10 |
|  | CA | CC | 0.59 | -0.39 | 0.68 |
|  |  | AA | 0.86 | -1.53 | 1.27 |
|  | AA | CC | 0.70 | -1.10 | 1.65 |
|  |  | CA | 0.86 | -1.27 | 1.53 |

| Weight | Genotype | Genotype | Significance | 95% Confidence Interval | |
| --- | --- | --- | --- | --- | --- |
|  |  |  |  | Lower Bound | Upper Bound |
| LSD | CC | CA | 0.93 | -0.19 | 0.21 |
|  |  | AA | 0.40 | -0.75 | 0.30 |
|  | CA | CC | 0.93 | -0.21 | 0.19 |
|  |  | AA | 0.38 | -0.77 | 0.30 |
|  | AA | CC | 0.40 | -0.30 | 0.75 |
|  |  | CA | 0.38 | -0.30 | 0.77 |

| Height | Genotype | Genotype | Significance | 95% Confidence Interval | |
| --- | --- | --- | --- | --- | --- |
|  |  |  |  | Lower Bound | Upper Bound |
| LSD | CC | CA | 0.67 | -0.01 | 0.01 |
|  |  | AA | 0.35 | -0.03 | 0.01 |
|  | CA | AA | 0.67 | -0.01 | 0.01 |
|  |  | AA | 0.45 | -0.02 | 0.01 |
|  | AA | CC | 0.35 | -0.01 | 0.03 |
|  |  | CA | 0.45 | -0.01 | 0.02 |
